# CD56+ immune cell infiltration and MICA are decreased in breast lobules with fibrocystic changes

**DOI:** 10.1007/s10549-017-4558-0

**Published:** 2017-11-01

**Authors:** Daniel Kerekes, Daniel W. Visscher, Tanya L. Hoskin, Derek C. Radisky, Rushin D. Brahmbhatt, Alvaro Pena, Marlene H. Frost, Muhammad Arshad, Melody Stallings-Mann, Stacey J. Winham, Linda Murphy, Lori Denison, Jodi M. Carter, Keith L. Knutson, Amy C. Degnim

**Affiliations:** 1 Department of Surgery, Mayo Clinic, 200 First Street SW, Rochester, MN 55905 USA; 2 Division of Anatomic Pathology, Mayo Clinic, Rochester, MN USA; 3 Division of Biomedical Statistics and Informatics, Mayo Clinic, Rochester, MN USA; 4 Cancer Biology, Mayo Clinic, Jacksonville, FL USA; 5 Women’s Cancer Program, Mayo Clinic, Rochester, MN USA; 6 Information Technology, Mayo Clinic, Rochester, MN USA; 7 Immunology, Mayo Clinic, Jacksonville, FL USA

**Keywords:** Natural killer cell, CD56, Activating ligand MICA, Benign breast disease

## Abstract

**Purpose:**

While the role of natural killer (NK) cells in breast cancer therapy has been investigated, little information is known about NK cell function and presence in nonmalignant and premalignant breast tissue. Here, we investigate and quantify NK cell marker CD56 and activating ligand MICA in breast tissue with benign breast disease.

**Methods:**

Serial tissue sections from 88 subjects, 44 with benign breast disease (BBD) who remained cancer-free, and 44 with BBD who later developed cancer, were stained with H&E, anti-MICA, and anti-CD56. Up to ten representative lobules were identified on each section. Using digital image analysis, MICA and CD56 densities were determined for each lobule, reported as percent of pixels in the lobule that registered as stained by each antibody. Analyses were performed on a per-subject and per-lobule basis.

**Results:**

Per-subject multivariate analyses showed associations of CD56 and MICA with age: CD56 was increased in older subjects (*p* = 0.03), while MICA was increased in younger subjects (*p* = 0.005). Per-lobule analyses showed that CD56 and MICA levels were both decreased in lobules with fibrocystic change, with median levels of CD56 and MICA staining, respectively, at 0.31 and 7.0% in fibrocystic lobules compared to 0.76 and 12.2% in lobules without fibrocystic change (*p* < 0.001 for each). Among fibrocystic lobules, proliferative/atypical lobules showed significantly lower expression compared to nonproliferative lobules for MICA (*p* = 0.02) but not for CD56 (*p* = 0.80).

**Conclusion:**

Levels of CD56+ NK cells and activating ligand MICA were decreased in breast lobules with fibrocystic change, and MICA levels showed a significant stepwise decrease with increasing histopathologic abnormality. MICA levels were also significantly decreased in older subjects, who generally have higher risk of developing cancer. These findings advance a model in which MICA promotes cytotoxic activity in CD56+ NK cells to protect against tumorigenesis in breast lobules, and suggest further research is warranted.

**Electronic supplementary material:**

The online version of this article (10.1007/s10549-017-4558-0) contains supplementary material, which is available to authorized users.

## Introduction

The past two decades have seen a substantial expansion in the proposed role of the immune system in protecting against carcinogenesis, outlined in various models ranging from immunosurveillance [1, 2] to immunoediting [3, 4] and tumor escape [5–7]. Current literature supports a role specifically for natural killer (NK) cells in immune-mediated protection against breast cancer, with increased presence of NK cells associated with an increase in effective breast tumor surveillance [8–14].

In the immunosurveillance hypothesis, epithelial cells with early DNA damage and stress may be recognized and cleared by the immune system, with NK cells playing a central role [15]. CD56, also known as neural cell adhesion molecule (NCAM), is a glycoprotein expressed on the surface of NK cells. CD56+ NK cells have been suggested to play a bifunctional role in early immune response through both highly cytotoxic behavior and upregulation of proinflammatory cytokine release [16].

MHC class I polypeptide-related sequence A (MICA) is an innate ligand for NKG2D, an activating receptor found on NK cells and T cells [17]. MICA may be a marker for early cellular stress, as it has been shown to be upregulated in conditions of heat shock [18] and oxidative stress [19]. MICA is largely absent in normal epithelium, but present in many epithelial tumors [20]. Once activated by MICA via NKG2D, NK cells have the ability to kill self-cells deficient in MHC class I molecules, a property important to immunosurveillance as tumor cells are known to evade the adaptive immune system through MHC class I downregulation [15].

To our knowledge, the presence of NK cells and their activating ligands in benign and premalignant human breast tissue has not been explored. We wished to explore the immunosurveillance role of NK cells in premalignant breast tissues by quantitating NK cell infiltrates and an activating marker of early cellular stress, MICA, in benign biopsy tissues from women who did and did not develop subsequent breast cancer. In this study, we carried out and quantified CD56 and MICA immunostaining in human breast tissues to investigate if and how CD56+ NK cell and NK cell ligand densities vary in nonmalignant breast lobules (1) between women who develop breast cancer and women who remain cancer-free, (2) according to age, (3) according to lobular involution, a histologic risk factor for cancer [21], and (4) according to fibrocystic status/epithelial proliferation, another established risk factor [22, 23].

## Methods

### Tissue samples

Approval was obtained from the Institutional Review Board to conduct this research. The investigation was carried out in a nested case–control design derived from the Mayo Clinic Benign Breast Disease (BBD) Cohort, a prospectively maintained cohort of > 13,000 women who underwent benign breast biopsy at Mayo Clinic between 1967 and 2001 [22]. Cases were women with BBD who subsequently developed breast cancer; controls had similar follow-up but did not develop breast cancer. Cases were matched to controls on age at biopsy, year of biopsy, and time to follow-up [24]. Fifty case–control pairs (100 tissue samples) were randomly selected for this intensive study. For each sample, serial tissue sections underwent the following stains: H&E, CD56, and MICA. Six of the 50 pairs had an inadequate CD56- or MICA-stained section and were excluded, leaving 44 pairs (88 samples) in the final analysis group.

### Histologic review

H&E-stained sections were reviewed by the study pathologist. Each sample was characterized for two “global” impressions of the entire slide: overall histologic impression and lobular involution, both of which are strongly associated with breast cancer risk [21, 22]. Histologic impression is based upon the highest degree of epithelial abnormality present within the sample, and is categorized as nonproliferative disease, proliferative disease without atypia, or atypical hyperplasia [22]. Age-related lobular involution was categorized as none (1–24% of normal lobules in tissue are involuted), partial (25–74%), or complete (≥ 75%) [21].

### Individual lobule annotation and histologic features

**Fig. 1:**
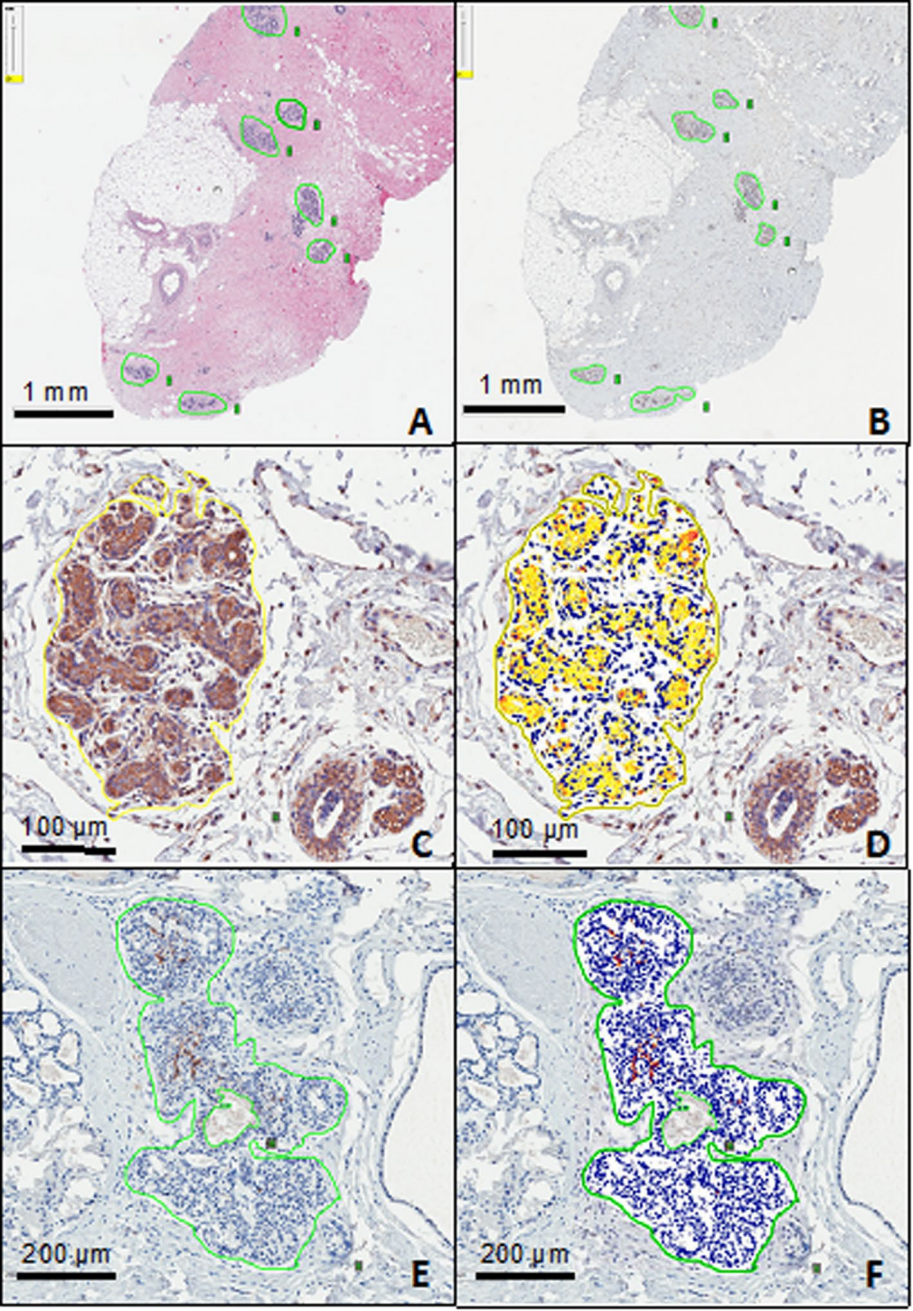
Histologic analysis of breast lobules. **a** H&E stain on a biopsy section, with representative lobules identified by pathologist. **b** MICA staining and lobule annotation on a serial section from the same biopsy as (**a**). **c** Annotation for analysis of a lobule stained for MICA. **d** Computer analysis run for MICA staining on lobule in (**c**). **e** Annotation for analysis of a lobule stained for CD56. **f** Computer analysis run for CD56 staining on lobule in (**e**). Analyses identify low-intensity MICA and CD56 reporter, moderate intensity, and strong intensity, in addition to normal cells, using yellow, orange, red, and blue highlights, respectively. Moderate intensity staining thresholds were used for all analyses

For each tissue sample, up to ten representative lobules were selected by the pathologist for detailed study (Fig. 1a). Each selected lobule was assigned a tracking number on the H&E stain, and the same lobules were annotated on the corresponding immunostained serial sections for quantification of CD56 and MICA staining. The pathologist characterized each individually numbered lobule on H&E stain as either normal or fibrocystic. Normal appearing lobules were further characterized individually as not involuted, partially involuted, or completely involuted. Fibrocystic lobules were sub-classified as nonproliferative, proliferative without atypia, or proliferative with atypia, according to the degree of epithelial proliferation. Photomicrographs of representative lobules at ×400 magnification were obtained using an Olympus 400 camera attached to a microscope.

### Immunohistochemical staining

Immunohistochemical staining procedures were performed as described previously [25]. CD56 immunostaining was performed with 1:25 dilution of DAKO M7304 Natural Killer Cell-NCAM, and MICA staining utilized 1:200 dilution of Abcam ab62540.

### Slide digitization and lobule annotation

Slide digitization and lobule annotation procedures were described previously [26]. By the methods outlined, whole slide digital images of all 264 breast biopsy tissue sections (H&E, anti-MICA, and anti-CD56 stains from each subject) were digitally scanned with the Aperio ScanScope AT2 slide scanner (Leica Biosystems, Buffalo Grove, IL) using the 20× objective lens. Boundaries of lobules were carefully marked for analysis and were considered to be the boundaries of the outermost epithelial cells in the lobule, with interlobular stroma and adipocytes excluded to the extent possible (Fig. 1b).

### Digital image analysis

After lobule annotation, levels of positive staining for CD56 and MICA were quantified using digital imaging software (Aperio Technologies). The manufacturer’s FDA-approved positive pixel count algorithms were used with optimization of the parameters for the CD56 and MICA stains [27]. The software uses specified threshold levels of pixel characteristics (Red Green Blue values and intensity levels) to identify and quantify positive staining levels and a digital color overlay to visually label pixels according to computed level of staining (Fig. 1c–f). The algorithm was applied uniformly to all annotated lobules across all samples. The algorithm calculated number of positively stained pixels and total number of pixels in each lobule analyzed. Levels of CD56 and MICA staining were calculated as a ratio of stained pixels to total pixels, in order to approximate the density of these markers throughout each lobule.

### Statistical analysis

Analyses were performed at both the per-subject and per-lobule level. To obtain per-subject estimates of MICA and CD56 percent positive pixel values, the medians within each subject were calculated across all lobules. Values for paired cases and controls were compared using Wilcoxon signed-rank tests. Other per-subject analyses to explore associations of MICA and CD56 with patient-level variables including age, global degree of lobular involution, and global histologic impression were performed using Kruskal–Wallis tests for univariate analysis and general linear models for multivariate analysis. At the per-lobule level, the correlation between the lobule-specific MICA and CD56 percent positive pixels was examined with scatter plots and estimated using Spearman’s rank correlation coefficient. Associations between lobule type and both MICA and CD56 values at the per-lobule level were assessed using linear mixed effects regression models with a random intercept for each subject to account for the correlation among multiple lobules from the same sample. Linear contrasts were used to test pairwise differences between lobule types within the mixed models. Due to a strong right skew, the Van der Waerden transformation was applied to MICA and CD56 percent values prior to modeling. The degree of within-sample variability across multiple lobules for MICA and CD56 was estimated by calculating a coefficient of variation (CV) for each sample as 100 × (within-sample SD/within-sample mean). Analysis was performed using SAS Version 9.3. *p* values < 0.05 were considered statistically significant.

## Results

### Characteristics of patients in analysis group

The median age of study subjects was 52 (range 35–73) at the time of biopsy (cases and controls were matched on age). The majority of biopsies were excisional (72%). Tissue samples exhibited a range of benign histologic findings [nonproliferative changes (36%), proliferative changes without atypia (43%), and atypical hyperplasia (21%)] and degree of lobular involution [no involution (25%), partial (41%), and complete (34%)]. These data are shown by case/control status in Table 1. The median time from biopsy to cancer in cases was 8.3 years (range 1.4–16.2 years), and the median length of follow-up in controls was 16.2 years (range 7.3–23.1 years). The cancers occurring among the 44 cases included 13 DCIS and 31 invasive cancers.

**Table 1:** Characteristics of patients with tissues included in BBD analysis presented by risk group

Variable	BBD case (*N* = 44)	BBD control (*N* = 44)	Total (*N* = 88)
Age at benign biopsy			
Median (range)	52 (35–73)	51.5 (36–73)	52 (35–73)
Age category			
< 45	10 (22.7%)	10 (22.7%)	20 (22.7%)
45–55	17 (38.6%)	16 (36.4%)	33 (37.5%)
> 55	17 (38.6%)	18 (40.9%)	35 (39.8%)
Biopsy type			
Core biopsy followed by excisional biopsy	1 (2.3%)	2 (4.5%)	3 (3.4%)
Core biopsy only	14 (31.8%)	8 (18.2%)	22 (25.0%)
Excisional biopsy only	29 (65.9%)	34 (77.3%)	63 (71.6%)
Histologic impression			
Nonproliferative	14 (31.8%)	18 (40.9%)	32 (36.4%)
Proliferative disease without atypia	19 (43.2%)	19 (43.2%)	38 (43.2%)
Atypical hyperplasia	11 (25.0%)	7 (15.9%)	18 (20.5%)
Lobular involution			
Not applicable*	3	0	3
None	12 (29.3%)	9 (20.5%)	21 (24.7%)
Partial	20 (48.8%)	15 (34.1%)	35 (41.2%)
Complete	9 (22.0%)	20 (45.5%)	29 (34.1%)

*For three samples, there were no normal lobules and therefore global evaluation of involution could not be assessed

### Characteristics of individual lobules

Among the 88 tissue samples, 770 lobules were annotated and studied (382 lobules in cases and 388 in controls). Of the 770 lobules, 43% were normal and 57% were fibrocystic. Of the 438 fibrocystic lobules, about half (51%) exhibited nonproliferative changes, 45% exhibited proliferative changes without atypia, and 4% exhibited atypical hyperplasia. Lobules demonstrated a range of involution, with 18% showing no involution, 39% showing partial involution, and 43% showing complete involution. These data as well as a breakdown by case status are presented in Table 2.

**Table 2:** Characteristics of lobules included in BBD analysis presented by risk group

Variable	BBD case (*N* = 382)	BBD control (*N* = 388)	Total (*N* = 770)
Lobule histologic impression			
Normal	140 (36.6%)	192 (49.5%)	332 (43.1%)
Fibrocystic	242 (63.4%)	196 (50.5%)	438 (56.9%)
Proliferative status among fibrocystic lobules			
Nonproliferative	118 (48.8%)	106 (54.1%)	224 (51.1%)
Proliferative	113 (46.7%)	84 (42.9%)	197 (45.0%)
Atypia	11 (4.5%)	6 (3.1%)	17 (3.9%)
Involution status among normal lobules			
None	24 (17.1%)	36 (18.8%)	60 (18.1%)
Partial	64 (45.7%)	65 (33.9%)	129 (38.9%)
Complete	52 (37.1%)	91 (47.4%)	143 (43.1%)

## Per-subject analyses

The first analyses performed were on the per-subject level in an attempt to uncover any associations between CD56+ cell infiltration, MICA staining, and features of age, global involution status, global histologic impression, and case–control status.

### Association of age and overall involution with MICA and CD56 levels

**Fig. 2:**
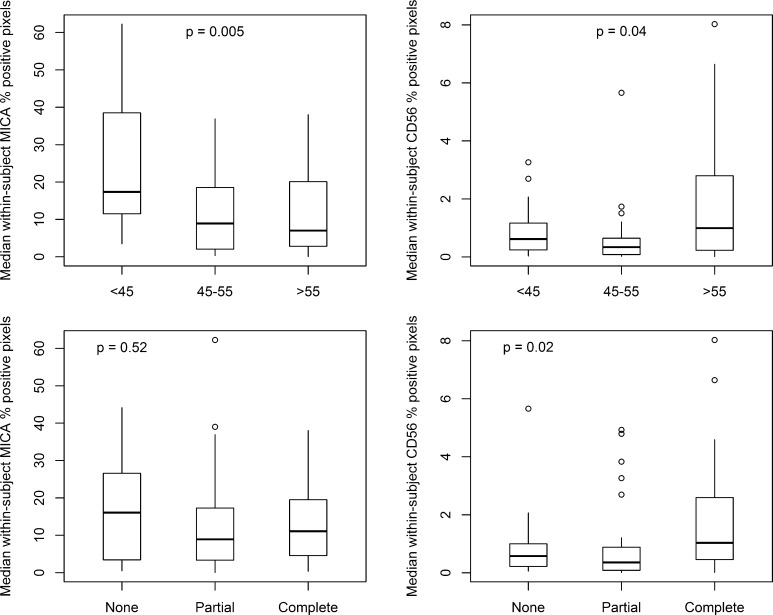
Per-subject median values for MICA and CD56 positive pixel percentages by age group and global degree of involution. *p* values are reported for univariate analyses. CD56 was significantly associated with both age and involution, while MICA was significantly associated with age only. MICA and CD56 show nearly opposite trends with respect to age. Top and bottom of boxes in plot represent 75th and 25th percentile values, respectively

Both MICA and CD56 showed a significant association (*p* = 0.005 and *p* = 0.04) with age at the per-subject level although in opposite directions (Fig. **2**): MICA was highest in younger women (< 45 years), while CD56 was highest in older women (> 55 years). MICA was not associated with global involution status, while CD56 was significantly higher for women with more complete involution (*p* = 0.02); however, this result represented confounding with age since CD56 was highest in women aged > 55, who are also those most likely to have complete involution. In a multivariate analysis including age, involution, histologic impression, and case status at the per-subject level, only age remained significantly associated with MICA (*p* = 0.005) and CD56 (*p* = 0.03).

### Global histologic impression and MICA/CD56

Both MICA and CD56 percent values were found to be highest in breast tissue samples exhibiting nonproliferative changes, with stepwise decreases in samples with proliferative epithelial changes without atypia and with atypical hyperplasia, but these differences were not significant (*p* = 0.64 and 0.85 for MICA and CD56, respectively). Median levels of MICA were 12.9% for nonproliferative changes, 9.9% for proliferative changes without atypia, and 9.2% for atypical hyperplasia; median values for CD56 for these groups were 0.64, 0.51, and 0.42%, respectively.

### Case status and MICA/CD56

Women who were BBD controls (no subsequent breast cancer) did not have statistically different overall tissue values of MICA nor CD56 as compared to BBD cases (patients that later developed cancer). Median MICA values were 10.0% in controls and 10.8% in cases (*p* = 0.70), whereas median CD56 values were 0.64% in controls and 0.39% in cases (*p* = 0.62).

## Per-lobule analyses

We also performed analyses on a per-lobule basis in order to further evaluate associations of MICA and CD56 with histologic features that vary for each lobule within a sample: fibrocystic status, degree of epithelial abnormality/proliferation, and involution status. Almost all lobules [98.1% (755/770)] showed presence of both CD56+ cells and MICA, and substantial within-subject variability was observed across multiple lobules from a single sample (median CV = 68% for MICA and median 114% for CD56, see Suppl Fig. 1 of MICA/CD56 plots on ten random samples). This variability across lobules provided additional rationale for performing per-lobule analyses.

### MICA and CD56 expression in fibrocystic versus normal lobules

**Fig. 3:**
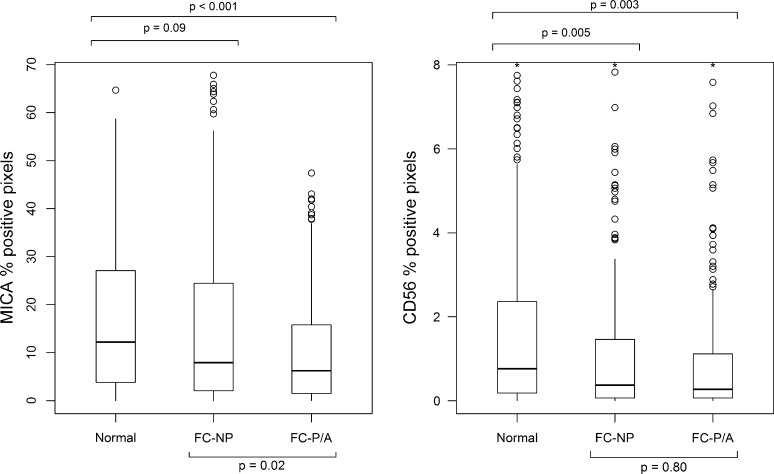
Per-lobule median values for MICA and CD56 positive pixel percentages by fibrocystic status, pairwise comparisons. Fibrocystic lobules were further clustered by proliferative status (*NP* nonproliferative changes, *P/A* proliferative changes without/or with atypia). *p* values were adjusted for subject age and case status. Top and bottom of boxes in plot represent 75th and 25th percentile values, respectively, and values beyond 8% pixels positive for CD56 were not included in the plot and were designated with asterisks. *p* values were < 0.001 for normal versus all fibrocystic lobules for both MICA and CD56

Compared to normal lobules, lobules that exhibited fibrocystic changes (with or without proliferation or atypia) had significantly lower median levels of both MICA (7.0 compared to 12.2%, *p* < 0.001) and CD56 (0.31 compared to 0.76%, *p* ≤ 0.001). Pairwise comparisons showed that compared to normal lobules, CD56 staining was significantly lower in fibrocystic nonproliferative lobules (*p* = 0.005) and fibrocystic proliferative/atypical lobules (*p* = 0.003). The same trend was observed for MICA, with higher expression in normal lobules, with the difference being significant compared to fibrocystic proliferative/atypia lobules (*p* < 0.001) but not significant compared to fibrocystic nonproliferative lobules (*p* = 0.02, Fig. 3).

### MICA and CD56 expression in fibrocystic proliferative versus nonproliferative lobules

When fibrocystic lobules were further classified by proliferative status into nonproliferative and proliferative/atypical, MICA showed lower levels in proliferative/atypical versus nonproliferative lobules (median 6.2% vs. 7.9%, *p* = 0.02), but CD56 did not (median 0.27% vs. 0.37%, *p* = 0.80, Fig. 3 **)**.

### MICA and CD56 by involution status in normal lobules

Among normal lobules classified as exhibiting no, partial, or complete involution, MICA and CD56 percent values were higher for greater degrees of involution, but these differences were not significant (*p* = 0.41 and 0.36 for MICA and CD56, respectively).

### Correlation of MICA and CD56 levels

At the per-lobule level, MICA and CD56 values showed a correlation of *r* = 0.28 for all lobules combined. This appears to be an age-dependent relationship, with only a weak correlation (*r* < 0.20) in those aged < 55, but a moderate correlation of *r* = 0.46 for women aged > 55 (Suppl Fig. 2). In women aged < 45, MICA seems high regardless of CD56 level, whereas for those aged > 55 MICA remains higher in women with higher CD56 and is lower in women with lower CD56.

## Discussion

MICA is a stress-inducible ligand that is expressed in many epithelial tumors [20]. It is an activating ligand for NKG2D, a receptor found on NK cells. When CD56+ NK cells are activated by MICA, they adopt a cytotoxic phenotype [17]. Through this mechanism, expression of MICA by premalignant cells in the absence of inhibitory signals promotes destruction by CD56+ NK cells before the mutated cells progress to cancer [28]. The current investigation quantitatively determined levels of CD56 and MICA expression in nonmalignant adult breast tissues with the hypothesis that lower levels of these markers would indicate suppressed NK cell immunoprotection in the breast epithelium and a higher incidence of cancer development.

In this study, both CD56 and MICA expressions were significantly lower in fibrocystic abnormal lobules compared to phenotypically normal lobules. Furthermore, MICA levels were lower in fibrocystic lobules with greater degree of abnormality. Higher CD56 levels and lower MICA levels were also each associated with older age, but had no association with involution status. Lastly, per-lobule MICA and CD56 expressions were positively correlated overall, with the strongest correlation in women over age 55.

Breast tissues with increasing degrees of fibrocystic change and epithelial abnormality are associated with a higher risk of developing breast cancer [22]. Therefore, the finding that lobules with fibrocystic changes show significantly lower levels of CD56 and MICA than normal lobules is consistent with the premise that decreased levels of MICA-activated CD56+ NK cells contribute to an environment of easier immune escape. It is possible that premalignant epithelial changes in the tissue are in part genetically characterized by decreased release of NK cell chemotactic factors and decreased expression of NK cell-activating ligands, such as MICA. The correlation of lobular MICA and CD56 levels strengthens a hypothesis in which MICA and CD56+ NK cells function together within an anti-tumor cytotoxic system. However, given the lack of direct association between these markers and an eventual cancer diagnosis, further studies are needed to confirm a functional role of MICA and CD56+ NK cells in tumor immunosurveillance.

Given the well-established association between age and breast cancer risk [29], we expected both CD56 and MICA presence to decrease with age. Consistent with our expectation, MICA levels significantly decreased with age; however, we found that CD56 levels were significantly increased in women of older age. If MICA and CD56 play a role in breast cancer immunosurveillance, it is possible that in older women, immunosurveillance underperformance stems predominantly not from a decrease in the density of CD56+ NK cells, but a decrease in the cytotoxicity of CD56+ NK cells as a result of decreased activation by MICA. Though CD56 expression levels are highest in the oldest subject group, MICA expression is lowest in this group. In fact, MICA demonstrates a significant (*p* = 0.005) stepwise decrease in expression with increasing age, mirroring the trend observed in degree of histopathologic abnormality. CD56 does not show as consistent or significant (*p* = 0.04) a trend across age groups, and disappears upon controlling for other factors.

In considering these findings, one particular strength of the current study is the use of human breast tissue ex vivo, as very few prior studies have evaluated NK cells in benign human breast tissues. Another important strength of this study was its ability to analyze staining levels by individual lobules, which comprise a spectrum of histologic abnormalities. In evaluating the results, it became clear that subject-level analyses were too broad to usefully reflect the physiology of immunosurveillance, as each tissue is a collection of lobules with varying biology. Other strengths include scalability of method, quantitative data on MICA and CD56 densities, and evaluation of two different markers for NK cell activity.

The primary limitation of this study is that we cannot infer functional immune cell activity from the immunohistochemical stains. We did not differentiate between CD56^dim^ and CD56^bright^ cells in this investigation, which have been reported to have different principal functions: CD56^dim^ cells appear to be primarily cytolytic in function, and CD56^bright^ cells primarily produce cytokines [16]. A second limitation of the study is that we did not find a direct association between either CD56 or MICA with risk of breast cancer in the per-subject analyses. Further limitations of the study include a relatively small number of women and lack of consideration for other risk factors including family history, BMI, life habits, and menopausal status. Lastly, we recognize that there are several described activating and inhibitory receptors on NK cells for which we did not stain in this study, [30] in addition to a number of other immune cells implicated in breast tumor surveillance [24].

## Conclusion

In summary, we found that CD56+ NK cells and MICA were present in benign breast tissues at significantly lower levels in lobules with epithelial abnormalities. Furthermore, CD56 expression was slightly positively correlated with age, while MICA expression was robustly negatively correlated with age. These findings could be explained by a hypothesis in which natural killer (NK) cells have an orchestrated cytotoxic functionality in the immunosurveillance of the normal breast that is compromised with age and in lobules with epithelial abnormalities. Specifically, these results could support a model in which proper MICA expression promotes CD56+ NK cell destruction of lobular cells that are cancerous or at risk for becoming cancerous. Further studies are necessary to refine the relationship between breast tissue histology and NK cell infiltration and to confirm a functional role of these cells in immunosurveillance.

## Electronic supplementary material

Below is the link to the electronic supplementary material.

Supplementary material 1 (DOC 315 kb)
