# Supplementary material for: CD56+ immune cell infiltration and MICA are decreased in breast lobules with fibrocystic changes
Source: Breast Cancer Res Treat. 2017 Nov 1;167(3):649–58. doi: 10.1007/s10549-017-4558-0 (PMC5807482; doi:10.1007/s10549-017-4558-0)

**Supplemental Figure 1.** Intra-sample variability across lobules for n=10 randomly selected women; asterisk denotes an outlier value.

**Supplemental Figure 2.** Plot of all lobules showing relative values of MICA and CD56 P^3^. A scattering of points along the y=x axis would suggest a perfect correlation between MICA and CD56 expression. A weak correlation is observed amongst all lobules, and a moderate correlation is observed amongst lobules from women >55. Spearman rank correlation coefficients (ρ) are reported. A constant (0.001) was added to MICA and CD56 values before taking the natural logarithm in order to preserve the lobules with 0 values in the plot.


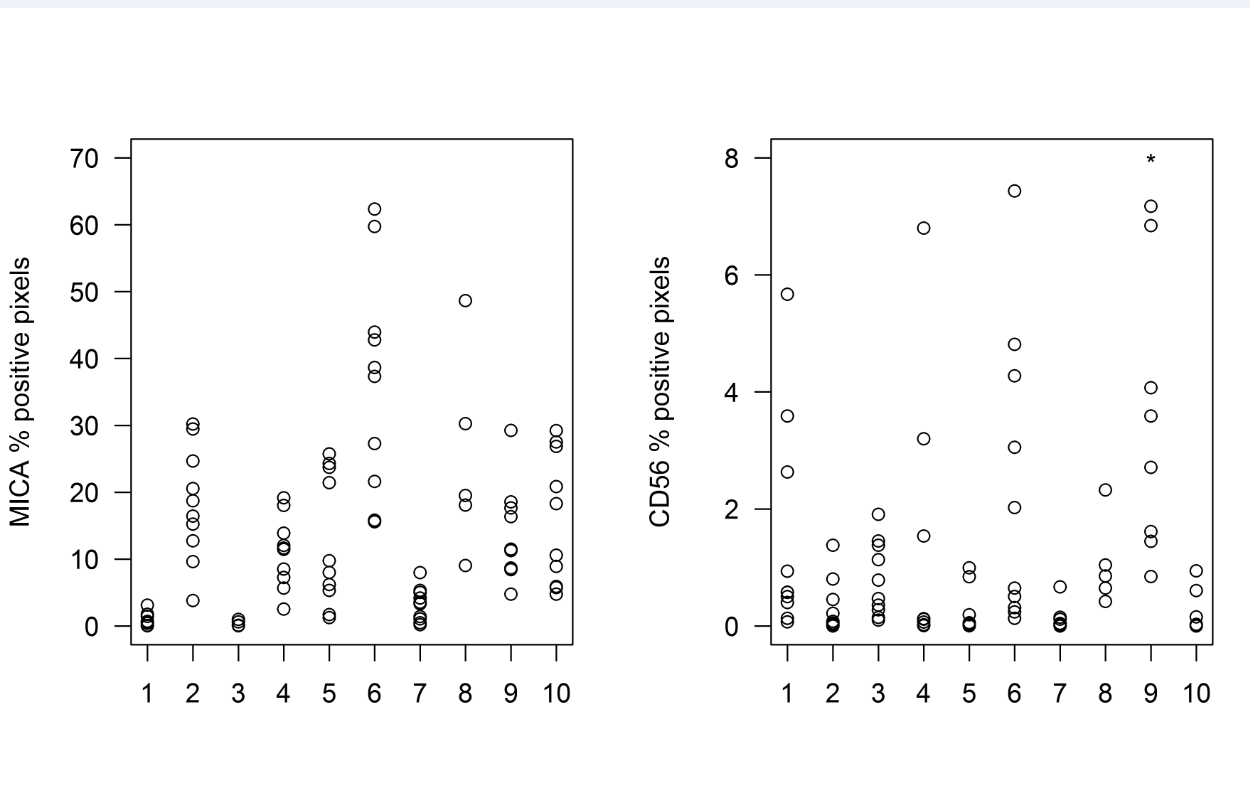

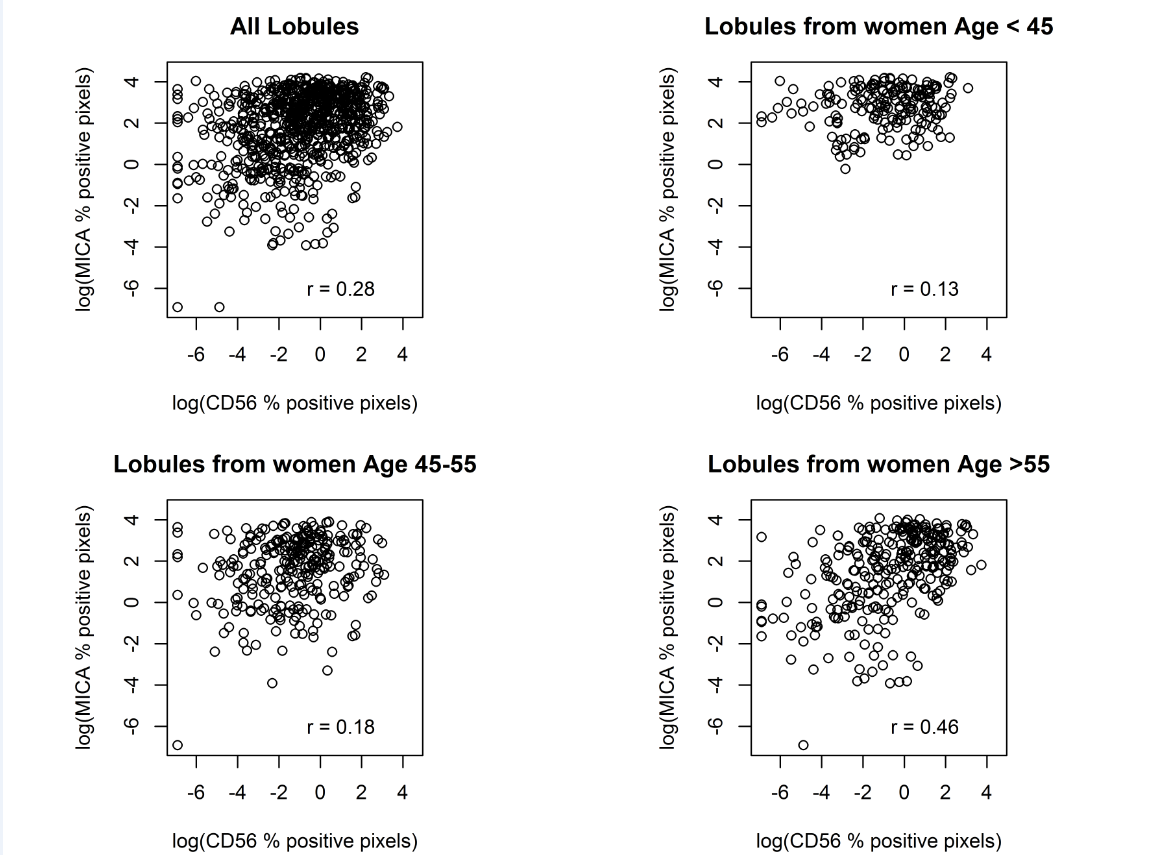

Supplement: Supplementary file 1 — Supplementary material 1 (DOC 315 kb) [file 10549_2017_4558_MOESM1_ESM.doc]
